# Research trends in the orthopedic surgical management of cerebral palsy: a cross-analytical study of publications in the past decade

**DOI:** 10.3389/fneur.2023.1200893

**Published:** 2023-08-22

**Authors:** Maher Ghandour, Matthias Klotz, Axel Horsch

**Affiliations:** ^1^Department of Orthopedics, Heidelberg University Hospital, Heidelberg, Germany; ^2^Marienkrankenhaus Soest, Orthopedics and Trauma Surgery, Soest, Germany

**Keywords:** cerebral palsy, cross-analytic, orthopedic, management, surgery

## Abstract

Little is known about the trends in orthopedic surgical management of cerebral palsy (CP). In this cross-analytical study we examined alterations in research publications in this field in the past 10 years through four databases. Thus, we divided publications into old (2012–2017) and recent (2018–2022). To determine if the focus of research in this field has changed, we compared both periods based on publication’s (authors’ number, journal, country, design), patients’ (number, gender, age, CP type), and surgery-related (indication, number, category, type) characteristics. Publications showed a positive trend over the past 10 years with a peak in 2020. The number of publications was similar between old and recent ones (47.58% vs. 52.42%). Most research outputs were from the United States and Germany. Differences were noted between recent and old publications regarding journals (*p* = 0.0001), journal category (*p* = 0.023), authors’ number (*p* = 0.006), and patients’ age (*p* = 0.02). The impact factor was also different (*p* = 0.0001). However, no differences were noted regarding other characteristics (*p* > 0.05). The research output regarding surgical orthopedic management in CP has increased in the past decade with no difference between 2012–2017 and 2018–2022. Except for the number of authors, journal name, and patients’ age, no significant differences were noted between both periods.

## 1. Introduction

Cerebral palsy (CP) is known as the leading cause of physical disability in children (1). It mainly affects children’s movement and posture, frequently manifesting during infancy and early childhood (2, 3). Although, to date, there is no conclusive evidence regarding the worldwide prevalence of CP, its rate tends to increase with increased patients’ survivorship into adulthood. In the United States (US), the estimated number of CP cases has been roughly estimated to range from 700,00 to 1,000,000 cases (4). A recent review indicated that CP occurs in 2–3 out of every 1,000 live childbirths (5).

Despite the fact that CP is characterized by non-progressive neuronal damage to the brain, the encountered musculoskeletal complications are frequent and they tend to worsen over time (6, 7). These complications include muscle weakness, abnormal muscle tone, which ultimately affect balance and coordinated movements. For example, these might include hip dysplasia, scoliosis, equinovarus foot, toe walking, torsional disorders, or fixed muscular contracture (8). These complications, along with hindered growth and developmental delays, negatively affect CP patients’ musculoskeletal system, leading to progressive contractures.

A wide variety of orthopedic surgeries have been implemented to correct such musculoskeletal deformities during the child’s growth as well as other deformities that might occur or persist into adulthood. The goal of orthopedic surgical management is to improve functional ambulation in ambulatory cases and to reduce the burden of care in non-ambulatory cases by preventing the experienced pain (9).

Evidence in the literature remains scarce regarding whether or not research on the orthopedic surgical management of CP has changed in the past 10 years. Our hypothesis is that the focus area of research on CP patients needing orthopedic surgical management has changed between old (defined as the period from late 2012 to late 2017) and recent (defined as the period from early 2018 to late 2022) publications. This potential change is proposed to have included not just the number of publications, but also patient (i.e., socioeconomic and clinical characteristics) and surgery (i.e., type of procedure) selection.

For this reason, we conducted this cross-analytical study to examine the changes in published research in this field between old and recent time periods. This study primarily aims to highlight if research conducted in the orthopedic surgical management of CP has declined throughout the past decade. Additionally, this research examines the focus area of published research in terms of publications’ characteristics, patients’ clinicodemographic data, and available surgical options.

## 2. Materials and methods

### 2.1. Study design and database search

This bibliometric cross-analytical study was conducted across four electronic databases (PubMed, Scopus, Web of Science, and Cochrane Library) on November 19, 2022. The systematic search was conducted as per the PRISMA guidelines and the PICOS framework. Meanwhile, the actual design, statistical analysis, and reporting followed the STROBE guidelines. The population included patients with CP (regardless of type), the intervention included any type or orthopedic surgeries, the comparison was not mandatory, the outcome was related to the year of publication (YOP), and the study design included all designs. Given the non-inclusion of human subjects in this research, the need for ethical approval by the Institutional Review Board was waived.

The systematic search was conducted through the use of several keywords and terms referring to “cerebral palsy” and “orthopedic surgery.” The detailed search strategy employed per each searched database is provided in Supplementary Table S1. Noteworthy, no filter was applied regarding language or study design; however, only records published in the past 10 years (Nov 2012 – Nov 2022) were retrieved and screened.

### 2.2. Inclusion/exclusion criteria

Records were included if they were: (1) published in the past 10 years, (2) included patients with CP, and (3) reported the conduct of any type of orthopedic surgery. Meanwhile, records were excluded if they had one of the following criteria: (1) duplicated records, (2) published before 2012, (3) non-human research, or (4) did not discuss orthopedic surgeries. Noteworthy, abstract-only papers were not excluded as long as they satisfied all of the above criteria.

### 2.3. Record selection

Following the retrieval of records, EndNote Software was used to remove duplicated records. Then, two independent reviewers screened all of the retrieved records through a combined screening strategy (both title/abstract and full-text screening). Full-text screening was only conducted when the abstract was not found to give a final decision whether to include or exclude a particular record. If both reviewers had different opinions regarding the inclusion of records, the senior author was consulted to give a final decision.

### 2.4. Study outcomes

The primary outcome of this bibliometric analysis is to compare the number of published studies on the orthopedic surgical management of CP between two different time periods: old (late 2012 – late 2017) and recent (early 2018 – late 2022). The secondary outcomes included the comparison between both timepoints in terms of publication characteristics, patients’ characteristics, and surgical information. Data on publication characteristics included the year of publication, the number of authors, journal name, journal category, journal impact factor, country, and study design. Noteworthy, the definition of each journal category and the estimation of each journal’s impact factor was done per the recent journal citation reports (JCR-2022) – Web of Science. Patients’ characteristics included the number of included CP patients in each record, age (categorized into: children, adults, children and adults, etc.), gender (categorized into: male only, female only, mixed), and type of CP (spastic, etc.). Surgical characteristics included the number of performed surgeries (accounting for laterality), the indication for each surgery, the name and the category of the surgical procedure, the affected body part (i.e., foot, ankle, hand, etc.), and the report of single-event multi-level surgery (SEMLS). Orthopedic surgeries were classified into six main categories: arthrodesis, muscle/ tendon lengthening, muscle/tendon shortening, osteotomy, tenotomy, or tendon transfer.

### 2.5. Data analysis

All of the retrieved data were analyzed using STATA Software (Version 17). Dichotomous/categorical data were presented as numbers and percentages. Meanwhile, continuous data were presented as means and standard deviations (SDs) or median and interquartile range (IQR) in normally and non-normally distributed data, respectively. The normality of data distribution was ascertained by data visualization using histograms and the Shapiro–Wilk test. The two-sample t-test was used to measure the difference between two or more groups regarding a continuous outcome. Chi-square test was used to compare dichotomous variables between the two groups. One-way ANOVA was used to compare the impact factor in different journal categories. A *p*-value of 0.05 or less was used as the cut-off point for statistical significance.

## 3. Results

### 3.1. Database search results

The electronic database search yielded 2,467 records, out of which 1,006 were excluded through EndNote software. The records of 1,461 publications were screened (both title/abstract and full-text), revealing a total number of 433 eligible records for final analysis. The remaining records were excluded for the following reasons: duplicated records (*n* = 51), no report or separate data for CP patients (*n* = 208), no report of orthopedic surgery (*n* = 369), and non-human research (*n* = 400).

### 3.2. Characteristics of included studies

The characteristics of included records is provided in Table 1. In summary, a total of 433 eligible records were identified. The mean number of authors was 4.83 (SD = 2.3), ranging from 1 to 17. Meanwhile, the median number of analyzed CP patients was 34 (IQR = 19–75), ranging from a single patient to as high as 3,305 patients. Overall, only 142 records reported the number of performed surgeries with a median value of 55 (IQR = 28–103) with a range of one to 1,088 surgeries.

**Table 1:** Summary statistics of the baseline characteristics of included records from the database search.

Variable	Number of publications	Mean	SD	Median	IQR	Range
Year of publication	433	2017.5	3.07	2018	2015–2020	2012–2022
Number of authors	433	4.83	2.3	5	3–6	1–17
Number of patients	316	82.02	228.87	34	19–75	1–3,305
Number of surgeries	142	111.57	171.1	55	28–103	1–1,088

SD, standard deviation; IQR, interquartile range.

**Figure 1:**
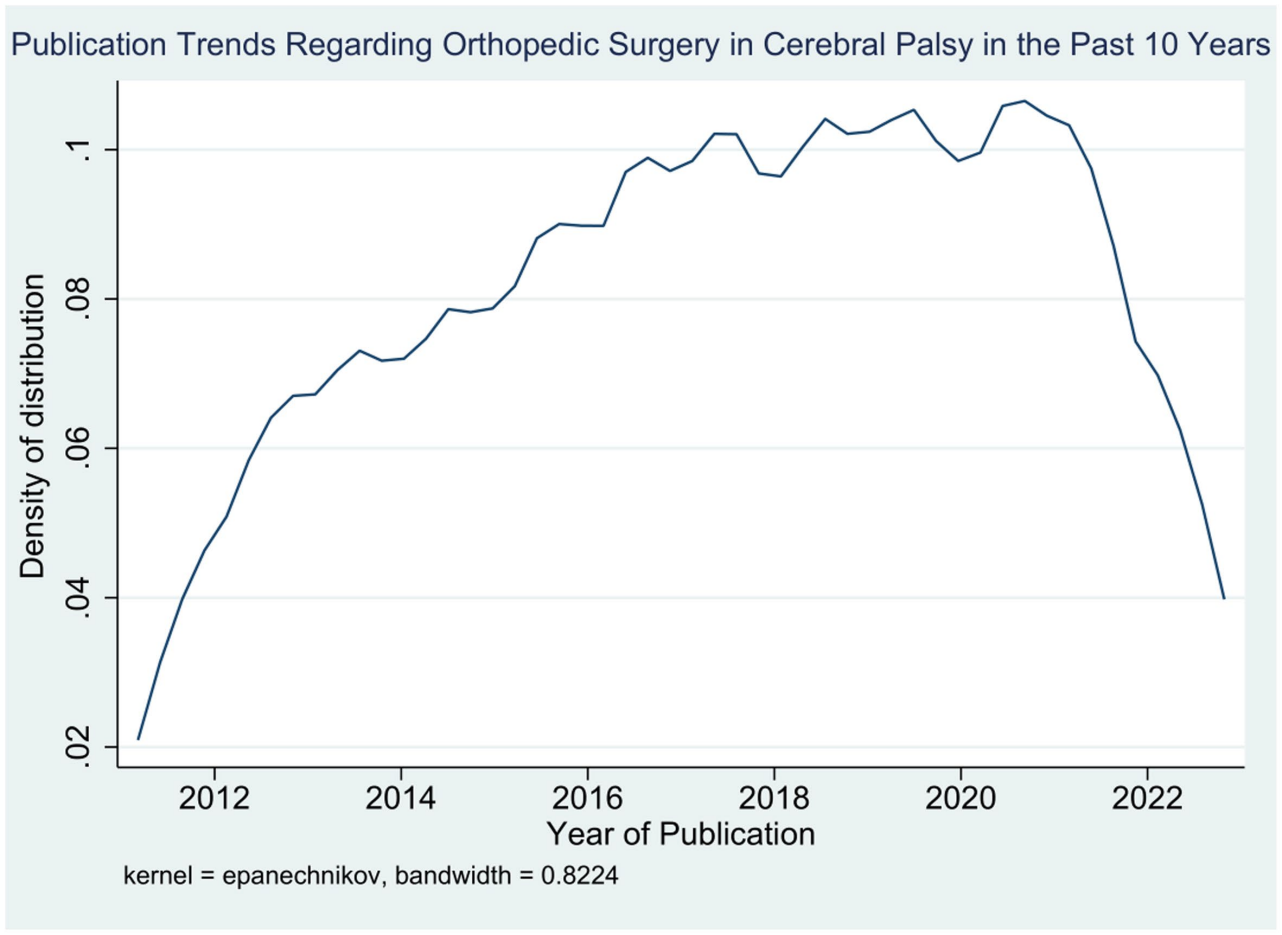
Trends in research output during the past 10 years on orthopedic surgical management of cerebral palsy.

Noteworthy, the number of published records was highest in 2020 (12.93%), right after the beginning of the COVID-19 pandemic, followed by 2022 (12.01%) and 2018 (11.55%), respectively. Meanwhile, the year 2014 accounted for the lowest number of publications (5.77%). A positive trend was noted in the number of records published per year during the past 10 years (Figure 1). Upon comparing the two time periods, it was noted that more research was published during 2018–2022 as compared to that published during 2012–2017 (52.42% vs. 47.58%) (Table 2).

**Table 2:** Summary statistics of the year of publication of retrieved records.

Variable	Category	N	T	%
Year of publication				
	2012	27	433	6.24%
	2013	35	433	8.08%
	2014	25	433	5.77%
	2015	38	433	8.78%
	2016	40	433	9.24%
	2017	41	433	9.47%
	2018	50	433	11.55%
	2019	34	433	7.85%
	2020	56	433	12.93%
	2021	35	433	8.08%
	2022	52	433	12.01%
Year of publication category				
	2012–2017	206	433	47.58%
	2018–2022	227	433	52.42%

N, number of records in each year; T, total number of records.

### 3.3. Publications characteristics

#### 3.3.1. Participating authors

The period (2018–2022) was associated with a significantly higher number of participating authors as compared to the previous period (2012–2017) with a mean value of 227 (SD = 0.14) vs. 206 (SD = 0.16) authors (*p* = 0.006).

#### 3.3.2. Publishing journals’ characteristics

**Figure 2:**
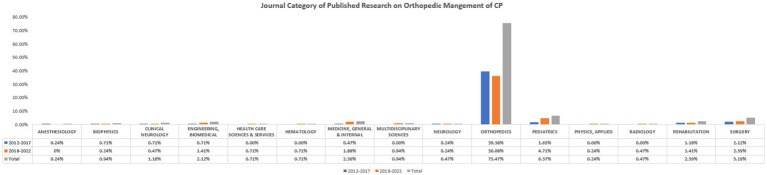
The number of publications per each journal category in the past 10 years.

In total, 118 journals have published research articles on orthopedic surgeries in CP during the past 10 years. A statistically significant difference in the frequency in which each journal published was noted between the two different time periods (2012–2017 vs. 2018–2022; *p* = 0.0001) (Supplementary Table S2). In terms of category, a total of 15 journal categories were identified (Figure 2). Orthopedic journals were ranked first (320/424; 75.47%) in publishing research on the surgical management of orthopedic conditions in CP patients followed by pediatric (27/424; 6.36%), surgery (22/424; 5.18%), and rehabilitation (11/424; 2.59%) journals, respectively. A statistically significant difference in the number of publications per journal between 2012–2017 and 2018–2022 was noted (*p* = 0.023) (Supplementary Table S2). The impact factor of different journal categories differed significantly (*p* < 0.00001).

Additionally, we categorized publishing journals into two categories: orthopedic and non-orthopedic journals. We observed a statistically significant difference in the number of publications between both categories (*p* = 0.003) (Table 3).

**Table 3:** The difference in the number of published articles per journal category according to impact factor.

Journal category	Journal IF		Total	*p*-value
	0–3	>3		
Non-orthopedic	29	16	45	0.003
Orthopedic	153	29	182	
Total	182	45	227	

IF, impact factor.

#### 3.3.3. Country of conducted research

**Figure 3:**
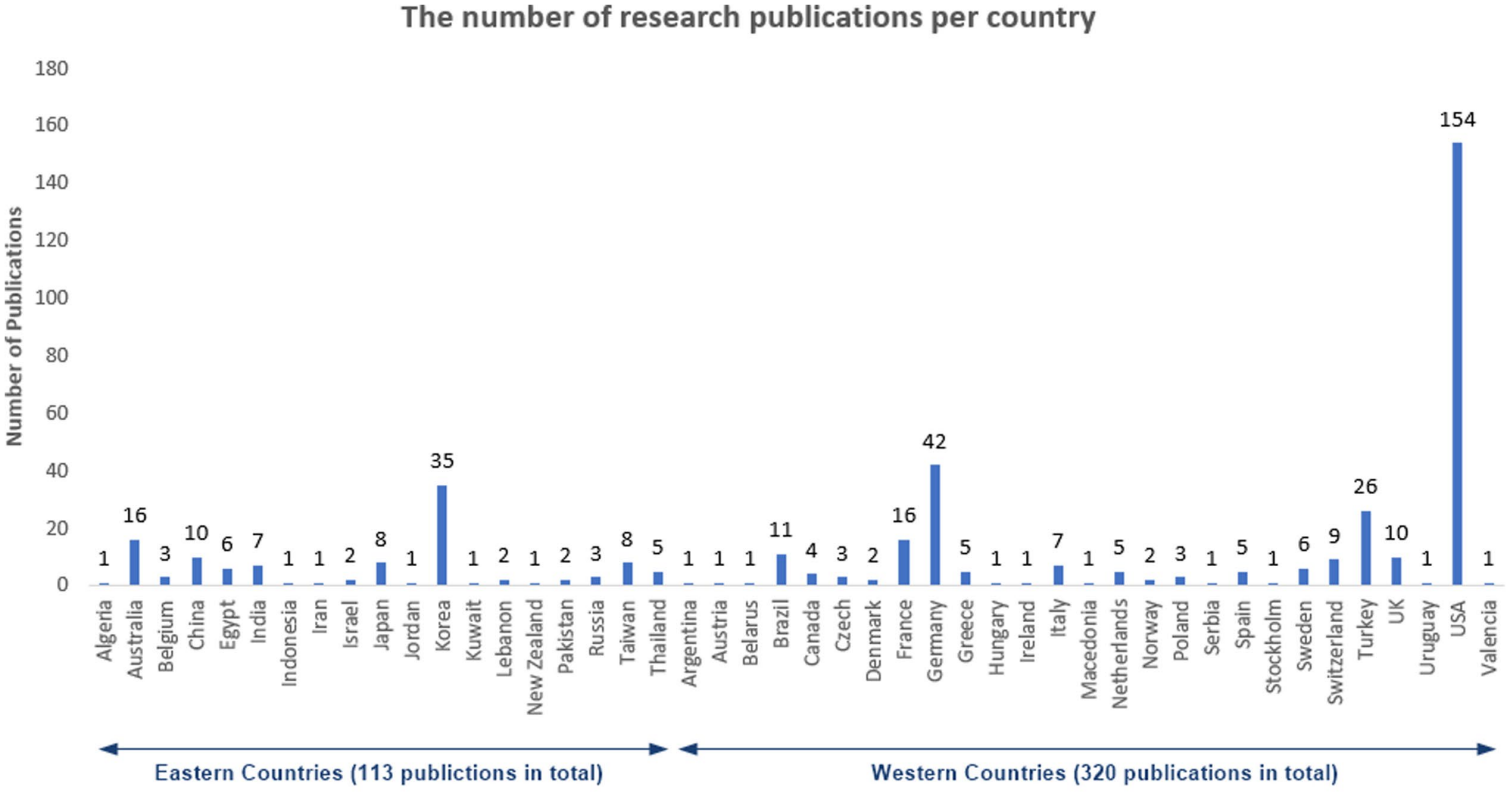
Distribution of records discussing orthopedic surgeries among cerebral palsy patients in the past 10 years.

Forty-six countries have published research on the orthopedic management of CP cases. Overall, the United States (35.57%) was the country with the highest number of publications, followed by Germany (9.7%), Korea (8.08%), and Turkey (6.0%), respectively (Figure 3). Upon comparing the number of publications per each country during 2012–2017 and 2018–2022, no statistically significant difference was noted (*p* = 0.265). Importantly, we observed that Western countries conducted and published CP-related research more than Eastern countries by almost three folds (320 vs. 113 articles, 283% percent difference), respectively.

#### 3.3.4. Study design of published reports

A total of 12 different study designs were published. The highest number of published records were retrospective cohort (62.73%) in design followed by systematic reviews and meta-analyses (14.35%). Noteworthy, only a few non-randomized (0.92%) and randomized clinical trials (1.62%) have been published in the past 10 years (Table 4). However, no statistically significant difference was noted in the number of published studies regarding study design during the two time periods (2012–2017 vs. 2018–2022; *p* = 0.434).

**Table 4:** The difference in the number of publications per study design during the past 10 years.

Design	YOP		Total
	2012–2017	2018–2022	
Retrospective cohort	130	141	271
Prospective cohort	13	11	24
Non-randomized trial	3	1	4
Randomized controlled trial	3	4	7
SR/MA	30	32	62
Commentary	4	9	13
Cross-sectional	0	3	3
Case series	11	16	27
Census	0	2	2
Case report	10	7	17
Protocol	1	0	1
Editorial	0	1	1
Total	205	227	432

YOP, year of publication.

### 3.4. Patients’ characteristics (number, age, gender, and type of CP)

A total of 316 records reported the number of included CP patients (Table 5). No statistically significant difference was noted in the number of included CP patients between 2012–2017 and 2018–2022 (mean difference = −32.55; 95% confidence interval: −81.41: 16.29; *p* = 0.190).

**Table 5:** The difference in cerebral palsy patients’ characteristics based on the year of publication.

Variable	Category	YOP		Total	*P*-value
		2012–2017	2018–2022		
Number of patients					
	Mean	64.61224	97.1716		0.19
	SD	150.2036	279.5		
	Observations	147	169	316	
Age					
	Adults	12	4	16	**0.02**
	Cadavers	1	0	1	
	Children	135	158	293	
	Children and adults	5	1	6	
	Children and young adults (<25 years)	3	9	12	
	Total	156	172	328	
Gender					
	Female	0	1	1	0.512
	Male	2	1	3	
	Mixed	39	42	81	
	Total	41	44	85	
Cerebral palsy type					
	Not reported	141	168	309	0.201
	Spastic	65	59	124	
	Total	206	227	433	

YOP, year of publication.Bold values refer to statistically significant differences (*p* < 0.05).

A total of 328 publications reported the age of included CP patients (Table 5). Overall, children were the most common age group (89.32%). Noteworthy, a statistically significant difference was noted in the number of publications reporting different age groups of CP patients between 2012–2017 and 2018–2022 (*p* = 0.020).

Remarkably, only a limited number of publications reported the gender of recruited CP patients (85 out of 433 records). That being said, no statistically significant difference was noted in the number of records reporting the gender of CP patients between 2012–2017 and 2018–2022 (*p* = 0.512).

The majority of studies did not report any data regarding the type of CP among included patients (309 out of 433 records). Meanwhile, in the remaining records, the spastic type was the only reported type. No statistically significant difference was noted in the number of publications reporting the type of CP between 2012–2017 and 2018–2022 (*p* = 0.201).

### 3.5. The characteristics of the implemented orthopedic surgical methods

#### 3.5.1. The number of performed surgeries in CP patients

Less than half the studies (142 out of 433) reported the number of performed orthopedic surgeries on CP patients. Upon comparing the number of performed orthopedic surgeries between 2012–2017 and 2018–2022, no statistically significant difference was observed (mean difference = 7.833; 95% confidence interval − 51.22: 66.89; *p* = 0.793).

#### 3.5.2. The indication for orthopedic surgery in CP

A total of 255 publications reported the indications for orthopedic surgery among CP patients (Supplementary Table S3). Overall, fixed flexion contracture was the most commonly reported indication for orthopedic surgery in CP, accounting for 16.47% of analyzed records followed by hip dislocation (9.80%) and crouch gait (9.01%) respectively. Of note, no statistically significant difference was noted in the indication for orthopedic surgeries among CP patients between 2012–2017 and 2018–2022 (*p* = 0.916).

#### 3.5.3. The body part on which orthopedic surgery was performed

Among 333 analyzed records, the hips (45.64%) were the most commonly reported body part on which orthopedic surgery was performed followed by the feet (15.61%) (Supplementary Table S4). Meanwhile, the metatarsophalangeal joints and the neck were the least investigated body parts (0.30%). The comparison of the number of studies reporting different body parts operated upon in CP between 2012–2017 and 2019–2022 revealed no statistically significant difference (*p* = 0.527).

#### 3.5.4. The type/category of orthopedic surgery in CP

Six categories of orthopedic surgeries have been reported in the literature (Supplementary Table S5). Of which, osteotomy was the most commonly reported surgery (48.26%) and the least commonly reported surgery was shortening (2.52%) in CP. No significant difference was noted in the number of investigated surgery categories between 2012–2017 and 2018–2022 (*p* = 0.255). Furthermore, the conduct of SEMLS was minimally reported in the literature (44 out of 433 records). The difference in the number of records reporting the conduct of SEMLS was different between 2012–2017 and 2018–2022; however, this difference was marginally statistically significant (*p* = 0.053).

A total of 86 different orthopedic surgeries in CP have been reported in the literature. Overall, femoral derotation osteotomy (FDO) was the most commonly (10.45%) reported orthopedic surgery conducted among CP patients followed by Hamstring muscle lengthening (HML) (8.84%) and varus derotation osteotomy (VDRO) (7.50%) (Supplementary Table S5). However, no statistically significant difference was noted in the number of studies reporting different surgeries between 2012–2017 and 2018–2022 (*p* = 0.225).

## 4. Discussion

This is the first bibliometric study to determine the publication trends regarding the orthopedic management of musculoskeletal deformities encountered in CP patients. Overall, 433 studies were identified and analyzed. Our study notes a positive trend in the number of publications over the past 10 years with a peak in the number of published records in 2020. This corresponds with the peak of COVID-19 pandemic, during which research output increased drastically as compared to before the pandemic (10). Additionally, the number of publications was high in 2022 and 2017. The lowest number of publications occurred in 2017. On the other hand, we noted that research output in this regard was slightly higher in the period from 2018 to 2022 as compared to that from 2012 to 2017. However, this difference did not reach statistical significance, and thus, our hypothesis of a change in research output is rejected.

The US is the number one country with research output regarding the orthopedic surgical management of CP-related musculoskeletal disorders followed by Germany, Korea, and Turkey, respectively. This finding points out the scarcity of research in this regard in most world countries, which could be attributed to either the lack of research resources or the lack of surgical expertise to perform such surgeries in CP patients, given the fact that the prevalence of CP is not widely different across countries (11). However, it should be noted that Western countries dominate the field of CP research; we noted an increase in CP-related research output by approximately three folds as compared to Eastern countries. However, we found no significant difference in the number of research outputs per country between the two timepoints, which reflects that each country maintained a consistent research output throughout the years. Remarkably, a total of 117 journals have been identified to publish research papers on the surgical correction of CP-related orthopedic conditions. But, the number of published papers per journal differed significantly between 2012–2017 and 2018–2022. Moreover, we noted a significant difference in the number of published records in each journal category between both timepoints. The majority of journal categories had a significant increase in the number of publications during 2018–2022 except for orthopedics journals which showed a significant decline. Furthermore, the impact factor of each journal category differed significantly. The number of authors also increased significantly during the period 2018–2022. This goes in line with the literature, confirming a positive trend regarding the growth rate of modern science, overall (12).

One of the most important aspects in this bibliometric study is the design of analyzed publications. Of note, the majority of published articles were retrospective cohort in nature, and systematic reviews were second in line. This supports the trend of increased conduct and publication of systematic reviews over the past 10 years, where nearly 80 reviews are published each day (13). Remarkably, in the past 10 years, only 4 non-randomized (14–17) and 7 randomized-controlled trials (18–24) have been published on the surgical management of CP-related orthopedic conditions. This highlights the need for more robust evidence to be conducted in order to determine the superiority of one surgical technique over others in correcting some of the orthopedic deformities in CP patients. That being said, no statistically significant difference was noted in the design of published research between 2012–2017 and 2018–2022.

In terms of patients’ characteristics, we did not observe any statistically significant differences regarding patients’ number, gender, or type of CP between 2012–2017 and 2018–2022. However, it is important to mention that the majority of publications did not report data regarding patients’ gender (80.36% missing data) or type of CP (71.36% missing data). This is an important point to consider in future research given the fact that anecdotal evidence supports the role of gender on intervention selection and outcome in CP patients (25). On the other hand, we noted a significant difference in the age group of CP patients between the two analyzed timepoints, highlighting an increase in the number of included CP children during 2018–2022.

In our study, no significant differences were observed regarding orthopedic surgeries-related characteristics. This can be explained by the wide variability of orthopedic surgeries in CP patients, indications for surgery, or affected body part. Regarding indications for orthopedic surgery, fixed flexion contracture of the affected limb was the most common reason followed by hip dislocation, crouch gait, and equinus, respectively. In the literature, equinus has been reported as the most common orthopedic complication in CP patients with a prevalence rate of 93% ranging from 71 to 99% (26). Although these data are inconclusive given the substantial heterogeneity, we believe that our finding is not representative of the actual rates of indications for orthopedics surgeries in CP patients given the fact that 41.10% of records reported no data on the indication for surgery. The hip was the most commonly affected body part in CP patients throughout the past 10 years, with osteotomy being the number one investigated and performed orthopedic surgery in CP patients. However, no significant differences were noted between the two timepoints (2012–2017 vs. 2018–2022). Noteworthy, an overall number of 86 orthopedic surgeries have been reported to correct 29 deformities (i.e., equinus, femoral anteversion, hip displacement, scoliosis, etc.). This points out the need for more research to investigate the accuracy and safety of each of these surgical procedures, preferably in a prospective manner with large sample size and proper study design.

Although our study is the first to highlight trends in research outputs regarding the surgical management of orthopedic disorders among CP patients, our study has several limitations. The percentage of missing data in some of our variables (i.e., indication for surgery, patients’ gender) limits the conclusions that can be driven from such findings while recommending the focus of future research on these points. Additionally, our analysis was based on published records, and thus, non-published and grey literature were not included which could affect the analysis findings.

## 5. Conclusion

The research output regarding the surgical management of orthopedic conditions in CP has increased throughout the past 10 year with a peak in 2020. The research output did not differ between 2012–2017 and 2018–2022. Except for the number of authors, journal name, and patients’ age, no significant differences were noted between both periods in terms of publication, patients’, and surgery characteristics.

## Data availability statement

The datasets generated and/or analyzed during the current study are available from the corresponding author on reasonable request.

## Author contributions

MH, AH, and MK: conceptualization. MH: methodology, software, formal analysis, data curation, writing—original draft preparation, and visualization. MK and AH: validation and writing—review and editing. AH: investigation, supervision, and project administration. MK: resources. All authors have read and agreed to the published version of the manuscript.

## Funding

For the publication fee we acknowledge financial support by Deutsche Forschungsgemeinschaft within the funding programme “Open Access Publikationskosten” as well as by Heidelberg University.

## Conflict of interest

The authors declare that the research was conducted in the absence of any commercial or financial relationships that could be construed as a potential conflict of interest.

## Publisher’s note

All claims expressed in this article are solely those of the authors and do not necessarily represent those of their affiliated organizations, or those of the publisher, the editors and the reviewers. Any product that may be evaluated in this article, or claim that may be made by its manufacturer, is not guaranteed or endorsed by the publisher.
