# Supplementary material for: Research trends in the orthopedic surgical management of cerebral palsy: a cross-analytical study of publications in the past decade
Source: Front Neurol. 2023 Aug 22;14:1200893. doi: 10.3389/fneur.2023.1200893 (PMC10482432; doi:10.3389/fneur.2023.1200893)
Supplement: Supplementary file 1 [file Table_1.DOCX]

Supplementary Material

Research Trends in the Orthopedic Surgical Management of Cerebral Palsy: A Cross-Analytical Study of Publications in the Past Decade

Maher Ghandour, Matthias Klotz, Axel Horsch^*^

*** Correspondence:** Axel Horsch: axel.horsch@med.uni-heidelberg.de

# Supplementary Tables

**Supplementary Table 1.** The detailed search strategy used in each of the searched electronic databases

| Database | No. | Search Query | Results |
| --- | --- | --- | --- |
| PubMed | | | |
|  | #1 | “cerebral palsy”[tiab] OR "Cerebral Palsy"[Mesh] | 30982 |
|  | #2 | “muscle lengthening”[tiab] OR “tendon lengthening”[tiab] OR “tendon transfer”[tiab] OR tenotomy[tiab] OR myotomy[tiab] OR osteotomy[tiab] OR arthrodesis[tiab] OR “selective dorsal rhizotomy”[tiab] OR tenodesis[tiab] OR “tendon shortening”[tiab] OR tendon-lengthening[tiab] OR lengthening[tiab] OR shortening[tiab] | 130135 |
|  | #3 | #1 AND #2 | 1584 |
|  | Filter | Date: last 10 years | 694 |
| Scopus | | | |
|  | #1 | TITLE-ABS-KEY (cerebral palsy) | 45725 |
|  | #2 | TITLE-ABS-KEY (lengthening) OR TITLE-ABS-KEY (shortening) OR TITLE-ABS-KEY (“tendon transfer”) OR TITLE-ABS-KEY (tenotomy) OR TITLE-ABS-KEY (myotomy) OR TITLE-ABS-KEY (osteotomy) OR TITLE-ABS-KEY (arthrodesis) OR TITLE-ABS-KEY (“selective dorsal rhizotomy”) OR TITLE-ABS-KEY (tenodesis) | 237397 |
|  | #3 | #1 AND #2 | 2348 |
|  | Filter | Date: last 10 years | 1036 |
| Web of Science | | | |
|  | #1 | AB=”cerebral palsy” | 19706 |
|  | #2 | AB=lengthening OR AB=shortening OR AB=”tendon transfer” OR AB=tenotomy OR AB=myotomy OR AB=osteotomy OR AB=arthrodesis OR AB=”selective dorsal rhizotomy” OR AB=tenodesis | 232710 |
|  | #3 | #1 AND #2 | 1135 |
|  | Filter | Date: last 10 years | 628 |
| Cochrane Library | | | |
|  | #1 | "cerebral palsy" | 4871 |
|  | #2 | “muscle lengthening” OR “tendon lengthening” OR “tendon transfer” OR tenotomy OR myotomy OR osteotomy OR arthrodesis OR “selective dorsal rhizotomy” OR tenodesis OR “tendon shortening” OR tendon-lengthening OR lengthening OR shortening | 8575 |
|  | #3 | #1 AND #2 | 148 |
|  | Filter | Date: last 10 years | 109 |
|  |  | RCTs=89 , Reviews = 19, protocol =1 | |

**Supplementary Table 2.** The difference in the number of publications per journal type during the past 10 years

| Journal | YOP | | Total |
| --- | --- | --- | --- |
|  | **2012-2017** | **2018-2022** |  |
| ACTA CHIRURGIAE ORTHOPAEDICAE ET TRAUMATOLOGIAE CECHOSLOVACA | 2 | 1 | 3 |
| Acta Orthop Traumatol Turc | 1 | 1 | 2 |
| Acta Orthopaedica | 1 | 6 | 7 |
| Acta Orthopaedica Belgica | 0 | 2 | 2 |
| Acta Orthopaedica et Traumatologica Turcica | 0 | 3 | 3 |
| Acta Ortop Bras | 1 | 0 | 1 |
| Acta Ortop Mex | 0 | 1 | 1 |
| Acta ortopédica mexicana | 1 | 0 | 1 |
| Anesthesiology Clinics | 1 | 0 | 1 |
| Annals of Physical and Rehabilitation Medicine | 0 | 1 | 1 |
| ANZ JOURNAL OF SURGERY | 0 | 1 | 1 |
| ARCHIVES OF ORTHOPAEDIC AND TRAUMA SURGERY | 0 | 1 | 1 |
| BMC MUSCULOSKELETAL DISORDERS | 0 | 5 | 5 |
| Bone and Joint Journal | 6 | 3 | 9 |
| Child Neurol Open | 0 | 1 | 1 |
| CHILDREN-BASEL | 0 | 2 | 2 |
| CHILDS NERVOUS SYSTEM | 1 | 0 | 1 |
| Chinese Journal of Anatomy and Clinics | 0 | 1 | 1 |
| CHIRURGIA | 1 | 0 | 1 |
| CLINICAL BIOMECHANICS | 0 | 1 | 1 |
| CLINICAL ORTHOPAEDICS AND RELATED RESEARCH | 2 | 0 | 2 |
| CLINICS IN ORTHOPEDIC SURGERY | 3 | 1 | 4 |
| Computer methods in biomechanics and biomedical engineering | 2 | 0 | 2 |
| CUKUROVA MEDICAL JOURNAL | 0 | 1 | 1 |
| CUREUS | 0 | 2 | 2 |
| CURRENT OPINION IN PEDIATRICS | 1 | 0 | 1 |
| Current Orthopaedic Practice | 0 | 1 | 1 |
| Developmental Medicine and Child Neurology | 5 | 13 | 18 |
| DISABILITY AND REHABILITATION | 1 | 0 | 1 |
| EKLEM HASTALIKLARI VE CERRAHISI-JOINT DISEASES AND RELATED SURGERY | 0 | 1 | 1 |
| European journal of orthopaedic surgery & traumatology: orthopedie traumatologie | 0 | 1 | 1 |
| European Journal of Orthopaedic Surgery and Traumatology | 1 | 2 | 3 |
| EUROPEAN JOURNAL OF PAEDIATRIC NEUROLOGY | 1 | 0 | 1 |
| EUROPEAN JOURNAL OF PHYSICAL AND REHABILITATION MEDICINE | 0 | 1 | 1 |
| EUROPEAN SPINE JOURNAL | 1 | 0 | 1 |
| FOOT & ANKLE INTERNATIONAL | 4 | 1 | 5 |
| Foot and Ankle Specialist | 0 | 1 | 1 |
| Foot and ankle surgery | 0 | 4 | 4 |
| Foot Ankle Clin | 0 | 1 | 1 |
| FRONTIERS IN SURGERY | 0 | 1 | 1 |
| Gait and Posture | 23 | 14 | 37 |
| Genij Ortopedii | 1 | 1 | 2 |
| Hand Clinics | 1 | 5 | 6 |
| Hand Surgery and Rehabilitation | 0 | 2 | 2 |
| HIP INTERNATIONAL | 1 | 0 | 1 |
| HSS JOURNAL | 0 | 2 | 2 |
| Indian Journal of Orthopaedics | 1 | 1 | 2 |
| International Orthopaedics | 3 | 3 | 6 |
| J Orthop Case Rep | 0 | 1 | 1 |
| J Orthop Sci | 0 | 1 | 1 |
| J Pediatr Orthop | 0 | 4 | 4 |
| J Rehabil Med Clin Commun | 0 | 1 | 1 |
| J Rural Med | 0 | 1 | 1 |
| JBJS Case Connector | 1 | 0 | 1 |
| JBJS Essent Surg Tech | 1 | 0 | 1 |
| JBJS REVIEWS | 1 | 4 | 5 |
| Joint Diseases and Related Surgery | 0 | 1 | 1 |
| Journal Medical Libanais | 0 | 1 | 1 |
| JOURNAL OF APPLIED BIOMATERIALS & FUNCTIONAL MATERIALS | 1 | 0 | 1 |
| JOURNAL OF ARTHROPLASTY | 2 | 0 | 2 |
| Journal of Bone and Joint Surgery | 9 | 1 | 10 |
| Journal of Children's Orthopaedics | 20 | 9 | 29 |
| JOURNAL OF CLINICAL MEDICINE | 0 | 2 | 2 |
| Journal of Clinical Orthopaedics and Trauma | 0 | 4 | 4 |
| Journal of Clinical Pediatric Surgery | 0 | 1 | 1 |
| Journal of Foot and Ankle Surgery | 4 | 1 | 5 |
| Journal of Hand Surgery | 13 | 3 | 16 |
| JOURNAL OF ISTANBUL FACULTY OF MEDICINE-ISTANBUL TIP FAKULTESI DERGISI | 0 | 1 | 1 |
| Journal of Multidisciplinary Healthcare | 0 | 1 | 1 |
| JOURNAL OF NEUROENGINEERING AND REHABILITATION | 2 | 0 | 2 |
| Journal of Orthopaedic Research | 1 | 0 | 1 |
| Journal of Orthopaedic Science | 1 | 2 | 3 |
| Journal of Orthopaedic Surgery | 0 | 1 | 1 |
| JOURNAL OF ORTHOPAEDIC SURGERY AND RESEARCH | 1 | 1 | 2 |
| Journal of Orthopaedics | 1 | 5 | 6 |
| Journal of Pediatric Orthopaedics | 44 | 47 | 91 |
| Journal of Pediatric Rehabilitation Medicine | 0 | 2 | 2 |
| JOURNAL OF SPINAL DISORDERS & TECHNIQUES | 1 | 0 | 1 |
| JOURNAL OF THE AMERICAN ACADEMY OF ORTHOPAEDIC SURGEONS | 1 | 2 | 3 |
| JOURNAL OF ULTRASOUND | 0 | 1 | 1 |
| Knee | 0 | 1 | 1 |
| Malaysian Orthopaedic Journal | 2 | 0 | 2 |
| Medeniyet Medical Journal | 0 | 1 | 1 |
| Medical Principles and Practice | 0 | 1 | 1 |
| MEDICINA-LITHUANIA | 0 | 2 | 2 |
| MEDICINE | 1 | 5 | 6 |
| Medicinski arhiv | 1 | 0 | 1 |
| Medicinski Glasnik | 0 | 1 | 1 |
| MINERVA ORTOPEDICA E TRAUMATOLOGICA | 0 | 1 | 1 |
| MINIMALLY INVASIVE SURGERY | 0 | 1 | 1 |
| Motricite Cerebrale | 1 | 0 | 1 |
| Musculoskeletal Surgery | 2 | 0 | 2 |
| Neurologic Clinics | 0 | 1 | 1 |
| NEUROREHABILITATION | 0 | 1 | 1 |
| OPERATIVE ORTHOPADIE UND TRAUMATOLOGIE | 0 | 1 | 1 |
| Operative Techniques in Orthopaedics | 3 | 0 | 3 |
| ORTHOPADE | 4 | 1 | 5 |
| Orthopaedics and Traumatology: Surgery and Research | 1 | 2 | 3 |
| ORTHOPEDIC CLINICS OF NORTH AMERICA | 0 | 1 | 1 |
| ORTHOPEDICS | 2 | 1 | 3 |
| Ortopedia Traumatologia Rehabilitacja | 1 | 0 | 1 |
| ORVOSI HETILAP | 0 | 1 | 1 |
| Paediatric Anaesthesia | 0 | 1 | 1 |
| PEDIATRIC ANESTHESIA | 1 | 0 | 1 |
| Pediatric Pelvic and Proximal Femoral Osteotomies: A Case-Based Approach | 0 | 2 | 2 |
| Pediatric Traumatology, Orthopaedics and Reconstructive Surgery | 3 | 1 | 4 |
| PLOS ONE | 0 | 3 | 3 |
| Prosthetics and Orthotics International | 0 | 1 | 1 |
| Research in Developmental Disabilities | 2 | 0 | 2 |
| Revista Espanola de Cirugia Ortopedica y Traumatologia | 0 | 1 | 1 |
| Seminars in Plastic Surgery | 2 | 0 | 2 |
| SICOT-J | 0 | 1 | 1 |
| Spine | 0 | 3 | 3 |
| TRAVMATOLOGIYA I ORTOPEDIYA ROSSII | 1 | 0 | 1 |
| Ultrasound in Medicine and Biology | 0 | 1 | 1 |
| WORLD JOURNAL OF PEDIATRICS | 1 | 0 | 1 |
| YONSEI MEDICAL JOURNAL | 1 | 0 | 1 |
| ZEITSCHRIFT FUR ORTHOPADIE UND UNFALLCHIRURGIE | 2 | 0 | 2 |
| Total | 201 | 223 | 424 |

YOP: year of publication

**Supplementary Table 3.** The difference in the indication for orthopedic surgery in CP based on the year of publication

| indication | YOP | | Total |
| --- | --- | --- | --- |
|  | **2012-2017** | **2018-2022** |  |
| Arthritis | 1 | 0 | 1 |
| Avascular Necrosis | 1 | 0 | 1 |
| Crouch Gait | 12 | 11 | 23 |
| Equinus | 10 | 12 | 22 |
| Equinocavovarus | 4 | 4 | 8 |
| Femoral anteversion | 6 | 4 | 10 |
| Foot drop | 0 | 2 | 2 |
| Flatfoot | 2 | 0 | 2 |
| Fixed Flexion Contracture | 17 | 25 | 42 |
| Fracture | 0 | 1 | 1 |
| Hip Displacement | 8 | 8 | 16 |
| Hip Dislocation | 13 | 12 | 25 |
| Dip Dysplasia | 10 | 10 | 20 |
| Hip instability | 1 | 2 | 3 |
| Hip internal rotation | 3 | 1 | 4 |
| Hip subluxation | 11 | 10 | 21 |
| Hallus valgus | 1 | 3 | 4 |
| Intoeing gait | 3 | 1 | 4 |
| Internal gait rotation | 6 | 5 | 11 |
| Knee Hyperextension | 1 | 1 | 2 |
| Planovalgus Foot Deformity | 9 | 12 | 21 |
| Painful Hip | 0 | 1 | 1 |
| Scoliosis | 1 | 0 | 1 |
| Spastic Hip disease | 0 | 1 | 1 |
| Stiff Knee Gait | 2 | 1 | 3 |
| Swan Neck | 1 | 0 | 1 |
| Spastic Wrist Flexion | 1 | 0 | 1 |
| Tibial Torsion | 1 | 1 | 2 |
| Valgus foot | 1 | 1 | 2 |
| Total | 126 | 129 | 255 |

YOP: year of publication

**Supplementary Table 4.** The difference in cerebral palsy patients’ affected body parts based on the year of publication

| Affected Part | YOP | | Total |
| --- | --- | --- | --- |
|  | **2012-2017** | **2018-2022** |  |
| Ankle | 6 | 8 | 14 |
| Elbow | 3 | 0 | 3 |
| Femur | 5 | 4 | 9 |
| Foot | 28 | 24 | 52 |
| Forearm | 2 | 0 | 2 |
| Hand | 4 | 3 | 7 |
| Hip | 72 | 80 | 152 |
| Knee | 17 | 32 | 49 |
| Lower limb | 7 | 9 | 16 |
| MTP | 0 | 1 | 1 |
| Neck | 1 | 0 | 1 |
| Spine | 5 | 4 | 9 |
| Tibia | 1 | 1 | 2 |
| Upper limb | 4 | 3 | 7 |
| Wrist | 5 | 4 | 9 |
| Total | 160 | 173 | 333 |

YOP: year of publication; MTP: Metatarsophalangeal joint

**Supplementary Table 5.** The difference in cerebral palsy patients’ surgery type/category based on the year of publication

| Variable | Category | YOP | | Total | P-value |
| --- | --- | --- | --- | --- | --- |
|  |  | **2012-2017** | **2018-2022** |  |  |
| Surgery Category | | | | | |
|  | Arthrodesis | 16 | 12 | 28 | 0.255 |
|  | Lengthening | 43 | 54 | 97 |  |
|  | Osteotomy | 77 | 76 | 153 |  |
|  | Shortening | 4 | 4 | 8 |  |
|  | Tenotomy | 7 | 6 | 13 |  |
|  | Tendon Transfer | 7 | 11 | 18 |  |
|  | Total | 154 | 163 | 317 |  |
| Single-Event Multi-Level Surgery | | | | | |
|  | Yes | 27 | 17 | 44s | 0.053 |
|  | No | 179 | 210 | 389 |  |
|  | Total | 206 | 227 | 433 |  |
| Surgery Type | | | | | |
|  | Arthrodesis | 16 | 11 | 27 | 0.225 |
|  | Anterior distal femoral hemiepiphysiodesis | 1 | 0 | 1 |  |
|  | Anterior Distal Femoral Plate Hemiepiphysiodesis | 0 | 1 | 1 |  |
|  | Ankle Foot Orthosis | 0 | 1 | 1 |  |
|  | Acetabuloplasty | 0 | 1 | 1 |  |
|  | Adductor-iliopsoas Tenotomy | 0 | 1 | 1 |  |
|  | Adductor Tenotomy | 0 | 2 | 2 |  |
|  | Braumann Procedure | 1 | 0 | 1 |  |
|  | Corrective Hip Surgery “not specified” | 0 | 1 | 1 |  |
|  | Calcaneal Lengthening | 7 | 4 | 11 |  |
|  | Calcaneal Lengthening Osteotomy | 1 | 2 | 3 |  |
|  | Calcaneal Osteotomy | 0 | 1 | 1 |  |
|  | Central Slip Tenotomy | 1 | 0 | 1 |  |
|  | Distal Femoral Extension Osteotomy | 2 | 7 | 9 |  |
|  | Distal Femoral Extension and Shortening Osteotomy | 1 | 1 | 2 |  |
|  | Distal Femoral Osteotomy | 1 | 0 | 1 |  |
|  | Distal Femoral Shortening Osteotomy | 0 | 1 | 1 |  |
|  | Distal Hamstring Lengthening | 1 | 1 | 2 |  |
|  | Dega Pelvic Osteotomy | 0 | 1 | 1 |  |
|  | Derotational Subtrochanteric Osteotomy | 0 | 1 | 1 |  |
|  | Dega Transiliac Osteotomy | 0 | 2 | 2 |  |
|  | Distal Tibia Rotation Osteotomy | 1 | 0 | 1 |  |
|  | Derotation Shortening Osteotomy | 1 | 0 | 1 |  |
|  | Epiphysiodesis | 0 | 1 | 1 |  |
|  | Elbow Muscle Lengthening | 1 | 0 | 1 |  |
|  | Flexor carpi ulnaris Transfer | 0 | 1 | 1 |  |
|  | Femoral derotation osteotomy | 21 | 18 | 39 |  |
|  | Fascia Lengthening | 1 | 0 | 1 |  |
|  | Femoral Osteotomy | 1 | 2 | 3 |  |
|  | Fusion surgery “not specified” | 1 | 1 | 2 |  |
|  | Femoral Valgus Osteotomy | 2 | 2 | 4 |  |
|  | Gastrocnemius Lengthening | 1 | 2 | 3 |  |
|  | Gastro-soleus Fascial Lengthening | 0 | 1 | 1 |  |
|  | Gastrocnemius Lengthening | 1 | 0 | 1 |  |
|  | Hemiepiphysiodesis | 1 | 0 | 1 |  |
|  | Hip fusion | 1 | 0 | 1 |  |
|  | Hamstring Muscle Lengthening | 11 | 22 | 33 |  |
|  | Hip Osteotomy | 1 | 0 | 1 |  |
|  | Hip Reconstruction “not specified” | 0 | 2 | 2 |  |
|  | Hip Reconstruction Surgery “not specified” | 1 | 4 | 5 |  |
|  | Reconstruction Surgery “not specified” | 2 | 10 | 12 |  |
|  | Intramuscular Psoas Lengthening | 1 | 0 | 1 |  |
|  | Lateral Column Lengthening | 0 | 1 | 1 |  |
|  | Muscle Lengthening “not specified” | 13 | 6 | 19 |  |
|  | Muscle Transfer | 1 | 0 | 1 |  |
|  | Osteotomy “not specified” | 12 | 8 | 20 |  |
|  | Periacetabular Osteotomy | 1 | 3 | 4 |  |
|  | 3-D printing acetabular plasty | 0 | 1 | 1 |  |
|  | Proximal Femoral Hemiepiphysiodesis | 0 | 1 | 1 |  |
|  | Plantar Flexor Lengthening | 0 | 1 | 1 |  |
|  | Proximal Femoral Osteotomy | 7 | 3 | 10 |  |
|  | Proximal Femoral Resection | 1 | 0 | 1 |  |
|  | Proximal Femoral Resection Arthroplasty | 1 | 0 | 1 |  |
|  | Proximal Femoral Screw Hemiepiphysiodesis | 0 | 1 | 1 |  |
|  | Proximal Femur Varus Osteotomy | 1 | 0 | 1 |  |
|  | Psoas Lengthening | 1 | 0 | 1 |  |
|  | Pronator Myotomy | 1 | 0 | 1 |  |
|  | Ponte Osteotomy | 1 | 0 | 1 |  |
|  | Percutaneous Pelvic Osteotomy | 1 | 1 | 2 |  |
|  | Posterior Spine Fusion | 1 | 0 | 1 |  |
|  | Percutaneous Subtrochanteric Osteotomy | 1 | 0 | 1 |  |
|  | Patella Lowering | 2 | 0 | 2 |  |
|  | Percutaneous Tenotomy and Aponeurotomy | 1 | 1 | 2 |  |
|  | Patellar Tendon Shortening | 3 | 2 | 5 |  |
|  | Percutaneous Tenotomy “not specified” | 3 | 0 | 3 |  |
|  | Rotational Osteotomy | 1 | 0 | 1 |  |
|  | Supracondylar Femoral Extension Osteotomy | 1 | 0 | 1 |  |
|  | Supracondylar Femoral Osteotomy | 1 | 0 | 1 |  |
|  | Salvage Hip Surgery | 1 | 2 | 3 |  |
|  | Split Anterior Tibialis Tendon Transfer | 1 | 1 | 2 |  |
|  | Selective Percutaneous Myofascial Lengthening | 0 | 3 | 3 |  |
|  | Soft Tissue Release | 0 | 2 | 2 |  |
|  | Subtrochanteric Valgus Osteotomy | 2 | 0 | 2 |  |
|  | Tenotomy | 3 | 5 | 8 |  |
|  | Tendo-Achilles Lengthening | 5 | 11 | 16 |  |
|  | Tibialis Anterior Tendon Shortening | 0 | 1 | 1 |  |
|  | Tibial Derotation Osteotomy | 3 | 2 | 5 |  |
|  | Total Hip Arthroplasty | 3 | 3 | 6 |  |
|  | Total Hip Replacement | 1 | 0 | 1 |  |
|  | Tendon Lengthening | 2 | 2 | 4 |  |
|  | Tendon Release and Lengthening | 1 | 0 | 1 |  |
|  | Tendon Release | 0 | 1 | 1 |  |
|  | Tendon Transfer | 6 | 9 | 15 |  |
|  | Varus derotational ostetomy | 10 | 18 | 28 |  |
|  | Varus derotational shortening ostetomy | 2 | 0 | 2 |  |
|  | Varus rotational osteotomy | 1 | 2 | 3 |  |
|  | Total | 177 | 196 | 373 |  |

YOP: year of publication
